# 2-(4,5-Dimeth­oxy-2-nitro­phen­yl)-4-meth­oxy-3-methyl-9-phenyl­sulfonyl-9*H*-carbazole

**DOI:** 10.1107/S1600536814003535

**Published:** 2014-02-22

**Authors:** P. Narayanan, K. Sethusankar, Velu Saravanan, Arasambattu K. Mohanakrishnan

**Affiliations:** a Department of Physics, RKM Vivekananda College (Autonomous), Chennai 600 004, India; b Department of Organic Chemistry, University of Madras, Maraimalai campus, Chennai 600 025, India

## Abstract

In the title compound, C_28_H_24_N_2_O_7_S, the carbazole system is essentially planar, with a maximum deviation of 0.0644 (19) Å for the C atom connected to the 4,5-dimeth­oxy-2-nitro­phenyl group. The dihedral angle between the carbazole moiety and the dimethoxy-substituted nitrophenyl ring is 58.55 (7)°. The sulfonyl group forms two intra­molecular C—H⋯O bonds with the adjacent carbazole system, forming two cyclic *S*(6) motifs. In the crystal, mol­ecules are linked along the *a* axis in bands consisting of cyclic *R*_3_^3^(15) motifs through two further C—H⋯O hydrogen bonds.

## Related literature   

For the biological activity and uses of carbazole derivatives, see: Itoigawa *et al.* (2000 ▶); Ramsewak *et al.* (1999 ▶). For their electronic properties and applications, see: Friend *et al.* (1999 ▶); Zhang *et al.* (2004 ▶). For related structures, see: Gopinath *et al.* (2013 ▶); Narayanan *et al.* (2014*a* ▶,*b* ▶). For the Thorpe–Ingold effect, see: Bassindale *et al.* (1984 ▶). For bond-length distortions, see: Allen *et al.* (1987 ▶). For graph-set notation, see: Bernstein *et al.* (1995 ▶).
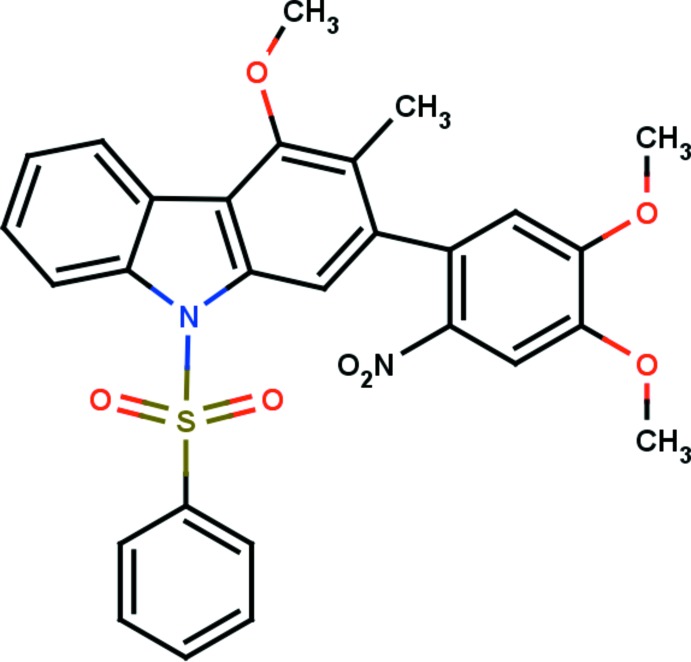


## Experimental   

### 

#### Crystal data  

C_28_H_24_N_2_O_7_S
*M* *_r_* = 532.56
Orthorhombic, 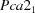

*a* = 8.4543 (3) Å
*b* = 13.6605 (5) Å
*c* = 21.6359 (9) Å
*V* = 2498.73 (16) Å^3^
*Z* = 4
Mo *K*α radiation
μ = 0.18 mm^−1^
*T* = 296 K
0.25 × 0.25 × 0.20 mm

#### Data collection  

Bruker Kappa APEXII CCD diffractometer
Absorption correction: multi-scan (*SADABS*; Bruker, 2008 ▶) *T*_min_ = 0.956, *T*_max_ = 0.964
14161 measured reflections
5152 independent reflections
4175 reflections with *I* > 2σ(*I*)
*R*_int_ = 0.028

#### Refinement  

*R*[*F* ^2^ > 2σ(*F* ^2^)] = 0.034
*wR*(*F* ^2^) = 0.085
*S* = 1.00
5152 reflections
347 parameters
1 restraint
H-atom parameters constrained
Δρ_max_ = 0.15 e Å^−3^
Δρ_min_ = −0.20 e Å^−3^

### 

Data collection: *APEX2* (Bruker, 2008 ▶); cell refinement: *SAINT* (Bruker, 2008 ▶); data reduction: *SAINT*; program(s) used to solve structure: *SHELXS97* (Sheldrick, 2008 ▶); program(s) used to refine structure: *SHELXL97* (Sheldrick, 2008 ▶); molecular graphics: *ORTEP-3 for Windows* (Farrugia, 2012 ▶) and *Mercury* (Macrae *et al.*, 2008 ▶); software used to prepare material for publication: *SHELXL97* and *PLATON* (Spek, 2009 ▶).

## Supplementary Material

Crystal structure: contains datablock(s) global, I. DOI: 10.1107/S1600536814003535/ld2120sup1.cif

Structure factors: contains datablock(s) I. DOI: 10.1107/S1600536814003535/ld2120Isup2.hkl

Supporting information file. DOI: 10.1107/S1600536814003535/ld2120Isup3.cml

CCDC reference: 987381

Additional supporting information:  crystallographic information; 3D view; checkCIF report

## Figures and Tables

**Table 1:** Hydrogen-bond geometry (Å, °)

*D*—H⋯*A*	*D*—H	H⋯*A*	*D*⋯*A*	*D*—H⋯*A*
C2—H2⋯O1	0.93	2.34	2.937 (3)	122
C11—H11⋯O2	0.93	2.34	2.939 (2)	122
C17—H17⋯O7^i^	0.93	2.55	3.372 (2)	148
C27—H27*A*⋯O3^ii^	0.96	2.59	3.167 (3)	119

Symmetry codes: (i) 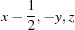; (ii) 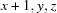.

**Fig. 1.**
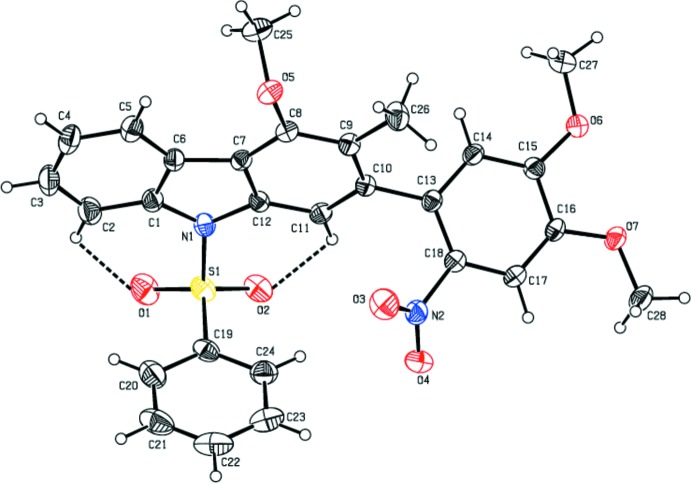
The molecular structure of the title compound with the atom numbering scheme, displacement ellipsoids are drawn at 30% probability level. H atoms are present as small spheres of arbitary radius. The intramolecular C—H···O hydrogen bonds, which are generate S(6) ring motifs, shown as a dashed lines (see Table 1 for details).

**Fig. 2.**
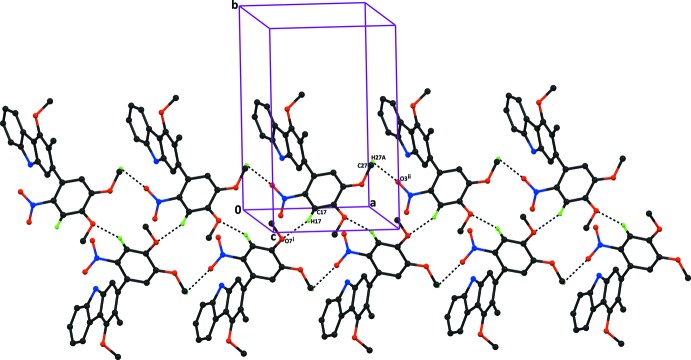
The packing arrangement of the title compound viewed down b axis. The dashed lines indicate C—H···O intermolecular interactions, which results in R33(15) ring motifs. The hydrogen atoms not involved in the hydrogen bonding and phenylsulfonyl group have been excluded for clarity. Symmetry codes: (i). -1/2 + x,-y,z (ii). 1 + x,y,z.

## supplementary crystallographic information

### 1. Comment

Carbazole and its derivative have become quite attractive compounds owing to their applications in pharmacy and molecular electronics. It has been reported that carbazole derivatives exhibit various biological activities such as antitumor (Itoigawa *et al.*, 2000), anti-inflammatory and antimutagenic (Ramsewak *et al.*, 1999). Carbazole derivatives also exhibit electroactivity and luminenscence and are considered to be potential candidates for electronic applications such as colour displays, organic semiconductors, laser and solar cells (Friend *et al.*, 1999;Zhang *et al.*, 2004).

The title compound, C_28_H_24_N_2_O_7_S, comprises a carbazole ring system which is attached to a phenylsulfonyl ring, a dimethoxy substituted nitrophenyl ring, a methoxy group and a methyl group. The carbazole ring system is essentially planar with maximum deviation of 0.0644 (19) Å for the carbon atom (C10). The methyl group carbon atom (C26) deviates from the adjacent carbazole ring by -0.172 (7) Å. The carbazole ring system is almost orthogonal to phenyl ring (C19–C24) attached to sulfonyl group with dihedral angle of 87.51 (10)°. The dihedral angle between the carbazole ring and the dimethoxy substituted nitrophenyl ring (C13–C18) is 58.55 (7)°.

The atom S1 has a distorted tetrahedral configuration. The widening of angle O2—S1—O1 [119.67 (11) °] and narrowing of angle N1—S1—C19 [105.07 (10)°] from the ideal tetrahedral value are attributed to the Thorpe-Ingold effect (Bassindale, *et al.* 1984). As a result of electron-withdrawing character of the phenylsulfonyl group, the bond lengths N1–C1 = 1.427 (3) Å and N1–C12 = 1.425 (3) Å in the molecule are longer than the mean value of 1.355 (14) Å (Allen, *et al.* 1987). The sum of the bond angles around N1 [356.2°] indicate the sp2 hybridization. The oxygen atoms O6 & O7 are deviated by 0.0347 (16) Å and -0.0240 (15) Å, respectively from the phenyl ring (C13–C18). The title compound exihibits the structural similarities with the already reported related stuctures (Gopinath *et al.* 2013; Narayanan *et al.* 2014*a*,*b*).

The molecular structure is stabilized by C2—H2···O1, C11—H11···O2 intramolecular interactions formed by sulfone oxygen atoms with carbazole moiety, which generate two S(6) ring motifs (Fig-1). In the crystal packing, molecules are linked by C17—H17···O7^i^ and C27—H27A···O3^ii^ intermolecular hydrogen bondings, which resulting in *R*_3_^3^(15) ring motifs (Bernstein, *et al.* 1995). The packing view of the title compound is shown in Fig-2. Symmetry codes: (i). -1/2 + *x*,-*y*,*z* (ii). 1 + *x*,*y*,*z*.

### 2. Experimental

A mixture of (*E*)-1-(2-(4,5-dimethoxy-2-nitrostyryl)-1- (phenylsulfonyl)-1H -indol-3-yl)-2-(phenylsulfonyl)propan-1-one (3.96 g, 6 mmol), dimethylsulfate (2.86 ml, 30 mmol) and K_2_CO_3_ (8.28 g, 60 mmol) in Tetrahydrofuran (100 ml) was stirred at room temperature for 18 h. After completion of the reaction (monitored by TLC), it was poured into crushed ice (100 g). The solid obtained was filtered and dried (CaCl_2_) to give enol ether. Then, the crued enol ether was dissolved in xylenes (100 ml) and refluxed for 24 h. Removal of xylenes *in vacuo* followed by column chromatographic purification (silica gel; hexane-ethyl acetate, 8:2) gave 9-(phenylsulfonyl)-2-(4,5-dimethoxy-2-nitrophenyl)-4-methoxy-3-methyl -9*H*-carbazole (2.23 g, 71%) as a colourless solid. Single crystals suitable for X-ray diffraction were prepared by slow evaporation of a solution of the title compound in chloroform (CHCl_3_) at room temperature.

m.p. 465–467 K.

### 3. Refinement

The positions of hydrogen atoms were localized from the difference electron density maps and their distances were geometrically constrained. The hydrogen atoms bound to the C atoms are treated as riding atoms, with d(C—H)=0.93 and *U*iso(H) = 1.2Ueq(C) for aromatic, d(C—H)=0.96 and *U*iso(H) =1.5*U*eq(C) for methyl groups. The rotation angles for methyl groups were optimized by least squares. In the absence of significant anomalous dispersion effects, an absolute structure was not determined and 1749 Friedel pairs were merged.

### Crystal data

**Table:**

C_28_H_24_N_2_O_7_S	*F*(000) = 1112
*M**_r_* = 532.56	*D*_x_ = 1.416 Mg m^−^^3^
Orthorhombic, *P**c**a*2_1_	Mo *K*α radiation, λ = 0.71073 Å
Hall symbol: P 2c -2ac	Cell parameters from 4175 reflections
*a* = 8.4543 (3) Å	θ = 2.4–27.2°
*b* = 13.6605 (5) Å	µ = 0.18 mm^−^^1^
*c* = 21.6359 (9) Å	*T* = 296 K
*V* = 2498.73 (16) Å^3^	Block, colourless
*Z* = 4	0.25 × 0.25 × 0.20 mm

### Data collection

**Table:**

Bruker Kappa APEXII CCD diffractometer	5152 independent reflections
Radiation source: fine-focus sealed tube	4175 reflections with *I* > 2σ(*I*)
Graphite monochromator	*R*_int_ = 0.028
ω and φ scans	θ_max_ = 27.2°, θ_min_ = 2.4°
Absorption correction: multi-scan (*SADABS*; Bruker, 2008)	*h* = −10→10
*T*_min_ = 0.956, *T*_max_ = 0.964	*k* = −10→17
14161 measured reflections	*l* = −27→27

### Refinement

**Table:**

Refinement on *F*^2^	Primary atom site location: structure-invariant direct methods
Least-squares matrix: full	Secondary atom site location: difference Fourier map
*R*[*F*^2^ > 2σ(*F*^2^)] = 0.034	Hydrogen site location: inferred from neighbouring sites
*wR*(*F*^2^) = 0.085	H-atom parameters constrained
*S* = 1.00	*w* = 1/[σ^2^(*F*_o_^2^) + (0.0466*P*)^2^ + 0.0209*P*] where *P* = (*F*_o_^2^ + 2*F*_c_^2^)/3
5152 reflections	(Δ/σ)_max_ < 0.001
347 parameters	Δρ_max_ = 0.15 e Å^−^^3^
1 restraint	Δρ_min_ = −0.20 e Å^−^^3^

### Special details

**Table:**

Geometry. All e.s.d.'s (except the e.s.d. in the dihedral angle between two l.s. planes) are estimated using the full covariance matrix. The cell e.s.d.'s are taken into account individually in the estimation of e.s.d.'s in distances, angles and torsion angles; correlations between e.s.d.'s in cell parameters are only used when they are defined by crystal symmetry. An approximate (isotropic) treatment of cell e.s.d.'s is used for estimating e.s.d.'s involving l.s. planes.
Refinement. Refinement of *F*^2^ against ALL reflections. The weighted *R*-factor *wR* and goodness of fit *S* are based on *F*^2^, conventional *R*-factors *R* are based on *F*, with *F* set to zero for negative *F*^2^. The threshold expression of *F*^2^ > σ(*F*^2^) is used only for calculating *R*-factors(gt) *etc*. and is not relevant to the choice of reflections for refinement. *R*-factors based on *F*^2^ are statistically about twice as large as those based on *F*, and *R*- factors based on ALL data will be even larger.

### Fractional atomic coordinates and isotropic or equivalent isotropic displacement parameters (Å^2^)

**Table:**

	*x*	*y*	*z*	*U*_iso_*/*U*_eq_	
C1	0.1268 (2)	0.39477 (15)	0.48958 (10)	0.0410 (5)	
C2	0.0620 (3)	0.41019 (19)	0.43157 (11)	0.0551 (6)	
H2	0.0564	0.3601	0.4026	0.066*	
C3	0.0063 (3)	0.5026 (2)	0.41856 (12)	0.0605 (7)	
H3	−0.0398	0.5146	0.3803	0.073*	
C4	0.0172 (3)	0.57813 (19)	0.46105 (12)	0.0550 (6)	
H4	−0.0196	0.6401	0.4505	0.066*	
C5	0.0817 (2)	0.56294 (17)	0.51863 (11)	0.0464 (5)	
H5	0.0879	0.6137	0.5472	0.056*	
C6	0.1375 (2)	0.46967 (16)	0.53321 (9)	0.0368 (5)	
C7	0.2083 (2)	0.42888 (14)	0.58853 (9)	0.0335 (4)	
C8	0.2422 (2)	0.46675 (13)	0.64640 (10)	0.0340 (4)	
C9	0.2998 (2)	0.40758 (15)	0.69354 (9)	0.0377 (5)	
C10	0.3300 (2)	0.30846 (14)	0.68049 (9)	0.0348 (4)	
C11	0.3021 (2)	0.26937 (15)	0.62219 (9)	0.0363 (4)	
H11	0.3258	0.2043	0.6136	0.044*	
C12	0.2382 (2)	0.32938 (14)	0.57735 (9)	0.0340 (4)	
C13	0.3936 (2)	0.24103 (14)	0.72890 (9)	0.0352 (4)	
C14	0.5425 (2)	0.25771 (15)	0.75502 (9)	0.0383 (5)	
H14	0.5989	0.3134	0.7438	0.046*	
C15	0.6080 (2)	0.19307 (14)	0.79735 (10)	0.0372 (4)	
C16	0.5267 (2)	0.10765 (14)	0.81391 (9)	0.0356 (4)	
C17	0.3815 (2)	0.08873 (15)	0.78787 (10)	0.0380 (4)	
H17	0.3271	0.0316	0.7976	0.046*	
C18	0.3174 (2)	0.15556 (15)	0.74717 (9)	0.0365 (4)	
C19	−0.0262 (2)	0.16388 (16)	0.51846 (11)	0.0460 (5)	
C20	−0.1607 (3)	0.18574 (18)	0.48542 (16)	0.0630 (7)	
H20	−0.1538	0.2158	0.4470	0.076*	
C21	−0.3066 (3)	0.1621 (2)	0.51058 (19)	0.0755 (9)	
H21	−0.3986	0.1764	0.4889	0.091*	
C22	−0.3159 (3)	0.1178 (2)	0.5671 (2)	0.0778 (9)	
H22	−0.4144	0.1019	0.5834	0.093*	
C23	−0.1818 (3)	0.0967 (2)	0.59984 (16)	0.0728 (8)	
H23	−0.1896	0.0675	0.6385	0.087*	
C24	−0.0346 (3)	0.11877 (18)	0.57567 (13)	0.0558 (6)	
H24	0.0570	0.1036	0.5974	0.067*	
C25	0.3301 (3)	0.63070 (16)	0.63949 (13)	0.0551 (6)	
H25A	0.4234	0.6198	0.6638	0.083*	
H25B	0.2946	0.6969	0.6451	0.083*	
H25C	0.3539	0.6198	0.5967	0.083*	
C26	0.3229 (3)	0.44965 (18)	0.75693 (11)	0.0550 (6)	
H26A	0.4304	0.4713	0.7614	0.082*	
H26B	0.3004	0.4005	0.7874	0.082*	
H26C	0.2528	0.5042	0.7626	0.082*	
C27	0.8274 (3)	0.29669 (17)	0.81872 (13)	0.0578 (7)	
H27A	0.8500	0.3075	0.7758	0.087*	
H27B	0.9244	0.2960	0.8417	0.087*	
H27C	0.7607	0.3483	0.8338	0.087*	
C28	0.5132 (3)	−0.03112 (18)	0.87996 (12)	0.0595 (7)	
H28A	0.4161	−0.0079	0.8976	0.089*	
H28B	0.5748	−0.0632	0.9113	0.089*	
H28C	0.4903	−0.0766	0.8473	0.089*	
N1	0.1942 (2)	0.30801 (12)	0.51522 (8)	0.0408 (4)	
N2	0.1579 (2)	0.13389 (15)	0.72451 (9)	0.0471 (5)	
O1	0.1521 (2)	0.20292 (13)	0.42413 (8)	0.0671 (5)	
O2	0.27674 (18)	0.13467 (11)	0.51672 (8)	0.0541 (4)	
O3	0.06190 (19)	0.20056 (13)	0.72205 (10)	0.0683 (5)	
O4	0.1284 (2)	0.04933 (13)	0.70995 (10)	0.0692 (5)	
O5	0.20795 (17)	0.56420 (10)	0.65879 (7)	0.0428 (4)	
O6	0.74881 (19)	0.20527 (10)	0.82589 (8)	0.0504 (4)	
O7	0.60017 (16)	0.04943 (10)	0.85569 (7)	0.0455 (4)	
S1	0.16035 (6)	0.19526 (4)	0.48928 (3)	0.04579 (14)	

### Atomic displacement parameters (Å^2^)

**Table:**

	*U*^11^	*U*^22^	*U*^33^	*U*^12^	*U*^13^	*U*^23^
C1	0.0372 (10)	0.0503 (12)	0.0357 (10)	−0.0021 (9)	0.0023 (10)	0.0052 (11)
C2	0.0557 (14)	0.0690 (17)	0.0406 (12)	−0.0028 (12)	−0.0034 (11)	0.0012 (12)
C3	0.0546 (15)	0.083 (2)	0.0439 (14)	0.0040 (12)	−0.0075 (11)	0.0171 (14)
C4	0.0493 (13)	0.0633 (15)	0.0525 (14)	0.0125 (11)	0.0033 (11)	0.0211 (13)
C5	0.0378 (11)	0.0542 (14)	0.0471 (12)	0.0084 (10)	0.0046 (10)	0.0099 (11)
C6	0.0289 (9)	0.0470 (13)	0.0345 (10)	0.0009 (8)	0.0033 (8)	0.0066 (9)
C7	0.0293 (9)	0.0342 (10)	0.0371 (10)	0.0004 (7)	0.0046 (8)	0.0038 (9)
C8	0.0322 (9)	0.0318 (10)	0.0378 (10)	0.0031 (8)	0.0068 (8)	−0.0018 (9)
C9	0.0407 (11)	0.0367 (12)	0.0357 (11)	0.0023 (8)	0.0015 (9)	−0.0015 (9)
C10	0.0359 (10)	0.0343 (11)	0.0342 (11)	0.0013 (8)	−0.0005 (8)	0.0014 (8)
C11	0.0399 (10)	0.0294 (10)	0.0395 (11)	0.0014 (8)	0.0030 (9)	−0.0018 (9)
C12	0.0330 (9)	0.0391 (11)	0.0297 (10)	−0.0012 (8)	0.0014 (8)	−0.0022 (9)
C13	0.0400 (10)	0.0325 (10)	0.0330 (10)	0.0026 (8)	0.0014 (8)	0.0006 (9)
C14	0.0471 (11)	0.0310 (11)	0.0368 (10)	−0.0018 (8)	0.0006 (9)	0.0035 (9)
C15	0.0360 (10)	0.0370 (11)	0.0386 (11)	0.0020 (8)	−0.0013 (9)	−0.0010 (9)
C16	0.0411 (10)	0.0308 (10)	0.0350 (11)	0.0065 (8)	0.0029 (9)	0.0049 (8)
C17	0.0417 (11)	0.0323 (10)	0.0400 (11)	0.0004 (8)	0.0051 (9)	0.0034 (9)
C18	0.0373 (10)	0.0363 (11)	0.0359 (11)	0.0014 (8)	0.0028 (8)	0.0000 (9)
C19	0.0444 (11)	0.0399 (12)	0.0537 (14)	−0.0001 (9)	−0.0046 (10)	−0.0154 (11)
C20	0.0592 (14)	0.0596 (15)	0.0703 (17)	0.0035 (12)	−0.0182 (14)	−0.0069 (14)
C21	0.0431 (14)	0.0681 (18)	0.115 (3)	−0.0007 (12)	−0.0155 (16)	−0.0148 (19)
C22	0.0527 (15)	0.0513 (16)	0.129 (3)	−0.0095 (12)	0.0142 (17)	−0.0140 (19)
C23	0.0725 (19)	0.0544 (16)	0.092 (2)	−0.0164 (14)	0.0137 (17)	0.0051 (15)
C24	0.0554 (13)	0.0466 (13)	0.0656 (16)	−0.0056 (11)	−0.0042 (12)	−0.0011 (12)
C25	0.0567 (14)	0.0394 (13)	0.0694 (17)	−0.0036 (10)	0.0125 (12)	−0.0006 (12)
C26	0.0780 (16)	0.0458 (14)	0.0410 (12)	0.0101 (11)	−0.0101 (12)	−0.0104 (11)
C27	0.0565 (14)	0.0532 (15)	0.0638 (16)	−0.0152 (11)	−0.0083 (12)	0.0003 (12)
C28	0.0570 (14)	0.0509 (14)	0.0706 (18)	0.0004 (11)	−0.0063 (12)	0.0275 (13)
N1	0.0461 (10)	0.0447 (10)	0.0317 (8)	−0.0019 (7)	−0.0011 (7)	−0.0021 (8)
N2	0.0430 (11)	0.0565 (13)	0.0418 (10)	−0.0035 (9)	0.0000 (8)	0.0088 (9)
O1	0.0784 (13)	0.0811 (13)	0.0417 (9)	−0.0006 (9)	0.0010 (9)	−0.0197 (9)
O2	0.0481 (8)	0.0516 (9)	0.0628 (10)	0.0097 (7)	−0.0031 (8)	−0.0177 (8)
O3	0.0396 (9)	0.0782 (13)	0.0870 (14)	0.0143 (8)	0.0000 (9)	0.0079 (11)
O4	0.0657 (11)	0.0574 (11)	0.0844 (14)	−0.0209 (9)	−0.0219 (10)	0.0096 (10)
O5	0.0463 (8)	0.0325 (8)	0.0497 (9)	0.0076 (6)	0.0090 (7)	−0.0014 (7)
O6	0.0490 (8)	0.0470 (9)	0.0553 (9)	−0.0077 (7)	−0.0142 (7)	0.0117 (7)
O7	0.0442 (8)	0.0384 (8)	0.0539 (9)	0.0018 (6)	−0.0056 (7)	0.0142 (7)
S1	0.0466 (3)	0.0508 (3)	0.0399 (3)	0.0019 (2)	−0.0005 (3)	−0.0144 (3)

### Geometric parameters (Å, º)

**Table:**

C1—C2	1.385 (3)	C18—N2	1.465 (3)
C1—C6	1.395 (3)	C19—C20	1.376 (3)
C1—N1	1.427 (3)	C19—C24	1.385 (3)
C2—C3	1.376 (4)	C19—S1	1.752 (2)
C2—H2	0.9300	C20—C21	1.386 (4)
C3—C4	1.385 (4)	C20—H20	0.9300
C3—H3	0.9300	C21—C22	1.366 (5)
C4—C5	1.376 (3)	C21—H21	0.9300
C4—H4	0.9300	C22—C23	1.368 (4)
C5—C6	1.395 (3)	C22—H22	0.9300
C5—H5	0.9300	C23—C24	1.383 (4)
C6—C7	1.449 (3)	C23—H23	0.9300
C7—C8	1.385 (3)	C24—H24	0.9300
C7—C12	1.404 (3)	C25—O5	1.437 (3)
C8—O5	1.389 (2)	C25—H25A	0.9600
C8—C9	1.389 (3)	C25—H25B	0.9600
C9—C10	1.407 (3)	C25—H25C	0.9600
C9—C26	1.500 (3)	C26—H26A	0.9600
C10—C11	1.390 (3)	C26—H26B	0.9600
C10—C13	1.495 (3)	C26—H26C	0.9600
C11—C12	1.380 (3)	C27—O6	1.423 (3)
C11—H11	0.9300	C27—H27A	0.9600
C12—N1	1.425 (3)	C27—H27B	0.9600
C13—C18	1.391 (3)	C27—H27C	0.9600
C13—C14	1.399 (3)	C28—O7	1.424 (3)
C14—C15	1.388 (3)	C28—H28A	0.9600
C14—H14	0.9300	C28—H28B	0.9600
C15—O6	1.351 (3)	C28—H28C	0.9600
C15—C16	1.401 (3)	N1—S1	1.6641 (18)
C16—O7	1.355 (2)	N2—O3	1.221 (2)
C16—C17	1.375 (3)	N2—O4	1.223 (2)
C17—C18	1.379 (3)	O1—S1	1.4152 (18)
C17—H17	0.9300	O2—S1	1.4163 (16)
			
C2—C1—C6	121.8 (2)	C24—C19—S1	118.54 (18)
C2—C1—N1	129.5 (2)	C19—C20—C21	118.7 (3)
C6—C1—N1	108.66 (18)	C19—C20—H20	120.6
C3—C2—C1	117.4 (2)	C21—C20—H20	120.6
C3—C2—H2	121.3	C22—C21—C20	120.4 (3)
C1—C2—H2	121.3	C22—C21—H21	119.8
C2—C3—C4	121.7 (2)	C20—C21—H21	119.8
C2—C3—H3	119.2	C21—C22—C23	120.6 (3)
C4—C3—H3	119.2	C21—C22—H22	119.7
C5—C4—C3	121.0 (2)	C23—C22—H22	119.7
C5—C4—H4	119.5	C22—C23—C24	120.3 (3)
C3—C4—H4	119.5	C22—C23—H23	119.9
C4—C5—C6	118.5 (2)	C24—C23—H23	119.9
C4—C5—H5	120.8	C23—C24—C19	118.8 (3)
C6—C5—H5	120.8	C23—C24—H24	120.6
C5—C6—C1	119.66 (19)	C19—C24—H24	120.6
C5—C6—C7	132.7 (2)	O5—C25—H25A	109.5
C1—C6—C7	107.68 (18)	O5—C25—H25B	109.5
C8—C7—C12	118.70 (17)	H25A—C25—H25B	109.5
C8—C7—C6	133.51 (18)	O5—C25—H25C	109.5
C12—C7—C6	107.72 (18)	H25A—C25—H25C	109.5
C7—C8—O5	119.31 (17)	H25B—C25—H25C	109.5
C7—C8—C9	121.26 (17)	C9—C26—H26A	109.5
O5—C8—C9	119.29 (18)	C9—C26—H26B	109.5
C8—C9—C10	118.44 (18)	H26A—C26—H26B	109.5
C8—C9—C26	119.60 (18)	C9—C26—H26C	109.5
C10—C9—C26	121.93 (19)	H26A—C26—H26C	109.5
C11—C10—C9	121.41 (18)	H26B—C26—H26C	109.5
C11—C10—C13	117.40 (17)	O6—C27—H27A	109.5
C9—C10—C13	121.19 (18)	O6—C27—H27B	109.5
C12—C11—C10	118.44 (18)	H27A—C27—H27B	109.5
C12—C11—H11	120.8	O6—C27—H27C	109.5
C10—C11—H11	120.8	H27A—C27—H27C	109.5
C11—C12—C7	121.64 (18)	H27B—C27—H27C	109.5
C11—C12—N1	130.06 (18)	O7—C28—H28A	109.5
C7—C12—N1	108.29 (16)	O7—C28—H28B	109.5
C18—C13—C14	116.04 (18)	H28A—C28—H28B	109.5
C18—C13—C10	123.34 (17)	O7—C28—H28C	109.5
C14—C13—C10	120.43 (17)	H28A—C28—H28C	109.5
C15—C14—C13	121.47 (18)	H28B—C28—H28C	109.5
C15—C14—H14	119.3	C12—N1—C1	107.51 (16)
C13—C14—H14	119.3	C12—N1—S1	123.55 (14)
O6—C15—C14	125.07 (18)	C1—N1—S1	124.66 (15)
O6—C15—C16	114.73 (17)	O3—N2—O4	123.89 (19)
C14—C15—C16	120.20 (19)	O3—N2—C18	118.41 (19)
O7—C16—C17	124.92 (18)	O4—N2—C18	117.69 (18)
O7—C16—C15	115.74 (17)	C8—O5—C25	113.57 (15)
C17—C16—C15	119.34 (18)	C15—O6—C27	118.00 (16)
C16—C17—C18	119.19 (19)	C16—O7—C28	117.58 (16)
C16—C17—H17	120.4	O1—S1—O2	119.67 (11)
C18—C17—H17	120.4	O1—S1—N1	106.02 (10)
C17—C18—C13	123.73 (19)	O2—S1—N1	106.26 (9)
C17—C18—N2	116.19 (19)	O1—S1—C19	109.43 (11)
C13—C18—N2	120.06 (17)	O2—S1—C19	109.35 (11)
C20—C19—C24	121.2 (2)	N1—S1—C19	105.07 (10)
C20—C19—S1	120.2 (2)		
			
C6—C1—C2—C3	−0.8 (3)	O7—C16—C17—C18	178.08 (18)
N1—C1—C2—C3	−179.3 (2)	C15—C16—C17—C18	−1.6 (3)
C1—C2—C3—C4	1.4 (3)	C16—C17—C18—C13	2.1 (3)
C2—C3—C4—C5	−1.3 (4)	C16—C17—C18—N2	−175.98 (17)
C3—C4—C5—C6	0.6 (3)	C14—C13—C18—C17	−0.8 (3)
C4—C5—C6—C1	−0.1 (3)	C10—C13—C18—C17	174.25 (19)
C4—C5—C6—C7	−178.9 (2)	C14—C13—C18—N2	177.23 (18)
C2—C1—C6—C5	0.1 (3)	C10—C13—C18—N2	−7.7 (3)
N1—C1—C6—C5	178.92 (17)	C24—C19—C20—C21	0.3 (4)
C2—C1—C6—C7	179.23 (18)	S1—C19—C20—C21	−178.4 (2)
N1—C1—C6—C7	−2.0 (2)	C19—C20—C21—C22	−0.1 (4)
C5—C6—C7—C8	1.7 (4)	C20—C21—C22—C23	0.4 (5)
C1—C6—C7—C8	−177.3 (2)	C21—C22—C23—C24	−1.0 (4)
C5—C6—C7—C12	178.6 (2)	C22—C23—C24—C19	1.1 (4)
C1—C6—C7—C12	−0.4 (2)	C20—C19—C24—C23	−0.8 (4)
C12—C7—C8—O5	−177.72 (16)	S1—C19—C24—C23	177.9 (2)
C6—C7—C8—O5	−1.1 (3)	C11—C12—N1—C1	177.16 (19)
C12—C7—C8—C9	−2.1 (3)	C7—C12—N1—C1	−3.8 (2)
C6—C7—C8—C9	174.50 (19)	C11—C12—N1—S1	19.6 (3)
C7—C8—C9—C10	2.8 (3)	C7—C12—N1—S1	−161.39 (14)
O5—C8—C9—C10	178.40 (17)	C2—C1—N1—C12	−177.8 (2)
C7—C8—C9—C26	−175.18 (19)	C6—C1—N1—C12	3.6 (2)
O5—C8—C9—C26	0.4 (3)	C2—C1—N1—S1	−20.5 (3)
C8—C9—C10—C11	−0.6 (3)	C6—C1—N1—S1	160.86 (15)
C26—C9—C10—C11	177.4 (2)	C17—C18—N2—O3	136.5 (2)
C8—C9—C10—C13	179.35 (18)	C13—C18—N2—O3	−41.7 (3)
C26—C9—C10—C13	−2.7 (3)	C17—C18—N2—O4	−42.5 (3)
C9—C10—C11—C12	−2.3 (3)	C13—C18—N2—O4	139.3 (2)
C13—C10—C11—C12	177.81 (17)	C7—C8—O5—C25	−84.7 (2)
C10—C11—C12—C7	3.0 (3)	C9—C8—O5—C25	99.6 (2)
C10—C11—C12—N1	−178.07 (19)	C14—C15—O6—C27	8.8 (3)
C8—C7—C12—C11	−0.8 (3)	C16—C15—O6—C27	−170.76 (19)
C6—C7—C12—C11	−178.28 (16)	C17—C16—O7—C28	−7.8 (3)
C8—C7—C12—N1	−179.98 (16)	C15—C16—O7—C28	171.89 (19)
C6—C7—C12—N1	2.6 (2)	C12—N1—S1—O1	−168.45 (17)
C11—C10—C13—C18	−57.7 (3)	C1—N1—S1—O1	37.77 (19)
C9—C10—C13—C18	122.3 (2)	C12—N1—S1—O2	−40.13 (18)
C11—C10—C13—C14	117.1 (2)	C1—N1—S1—O2	166.09 (17)
C9—C10—C13—C14	−62.8 (3)	C12—N1—S1—C19	75.72 (18)
C18—C13—C14—C15	−1.0 (3)	C1—N1—S1—C19	−78.07 (19)
C10—C13—C14—C15	−176.19 (19)	C20—C19—S1—O1	−24.4 (2)
C13—C14—C15—O6	−178.05 (19)	C24—C19—S1—O1	156.87 (19)
C13—C14—C15—C16	1.4 (3)	C20—C19—S1—O2	−157.29 (18)
O6—C15—C16—O7	−0.3 (3)	C24—C19—S1—O2	24.0 (2)
C14—C15—C16—O7	−179.81 (18)	C20—C19—S1—N1	89.0 (2)
O6—C15—C16—C17	179.45 (18)	C24—C19—S1—N1	−89.7 (2)
C14—C15—C16—C17	−0.1 (3)		

### Hydrogen-bond geometry (Å, º)

**Table:**

*D*—H···*A*	*D*—H	H···*A*	*D*···*A*	*D*—H···*A*
C2—H2···O1	0.93	2.34	2.937 (3)	122
C11—H11···O2	0.93	2.34	2.939 (2)	122
C17—H17···O7^i^	0.93	2.55	3.372 (2)	148
C27—H27*A*···O3^ii^	0.96	2.59	3.167 (3)	119

Symmetry codes: (i) *x*−1/2, −*y*, *z*; (ii) *x*+1, *y*, *z*.
